# Safety, Quality and Analytical Authentication of ḥalāl Meat Products, with Particular Emphasis on Salami: A Review

**DOI:** 10.3390/foods9081111

**Published:** 2020-08-13

**Authors:** Maria Martuscelli, Annalisa Serio, Oriana Capezio, Dino Mastrocola

**Affiliations:** 1 Faculty of Bioscience and Technology for Food, Agriculture and Environment, University of Teramo, Via R. Balzarini 1, 64100 Teramo, Italy; aserio@unite.it (A.S.); dmastrocola@unite.it (D.M.); 2 Department Asian, African and Mediterranean, University of Naples “L’Orientale”, Piazza San Domenico Maggiore 12, 80134 Napoli, Italy; ocapezio@unior.it

**Keywords:** ḥalāl salami, fermented sausages, ḥalāl assurance, authenticity

## Abstract

Only some animal species could be transformed into ḥalāl salami and the raw meat must be obtained from ritually slaughtered animals. Several scientific studies have been conducted on ritual slaughtering practices and manufacturing of meat products for Jewish and Muslim religious communities; furthermore, many projects have been funded by the European Community on this topic. The authenticity and traceability of meat is one of the priorities of ḥalāl food certification systems. The pig matrix (meat and/or lard) may be fraudulently present in ḥalāl processed meat, as well as salami, for both economic and technological purposes; in fact, the use of these raw materials reflects the easier availability and their lower cost; furthermore, it allows manufacturers to obtain final products with better quality (sensory properties) and stability (especially with respect to oxidative reactions). The aim of this review is to discuss the qualitative and technological aspects of ḥalāl raw meat for dry fermented sausages (salami); moreover, this study focuses on the most recent studies carried out on the certification system and on the analytical methods performed in order to solve problems such as fraud and adulteration of ḥalāl salami and other halal meat foods.

## 1. Ḥalāl Meat Products and Regulations in Europe

Today Muslim people constitute about 25% of the population in the world and this is expected to increase further; the size of the global ḥalāl market could reach around $2.6 trillion [1,2]. Muslims are projected to increase as a share of Europe’s population. The share of Muslims in Europe’s population as of 2050 would be depending on three possible future migration scenarios: it would be expected to be somewhere among 7.4% (zero migration) or between 11.2% (“regular” migration) and 14% (high migration) [3].

The halāl food market is a considerable economic opportunity for agro-food enterprises. The migratory flows and the substantial rise of the Muslim population in Europe not only affects the socio-cultural aspects but also from an economic point of view, regarding foodstuffs and other products (drugs, cosmetics, etc.) that must be permissible (ḥalāl) to Muslims consumers, following specific religious laws [4,5,6,7,8]. Malaysia has been the first country in the world to establish ḥalāl -related laws [9]; it has a documented and systematic ḥalāl assurance system; besides conventional legal texts, *fatwa* is a legal opinion issued by Islamic scholars based on interpretation and adaptation of verses from Koran and *Ahadith* [10].

**Figure 1:**
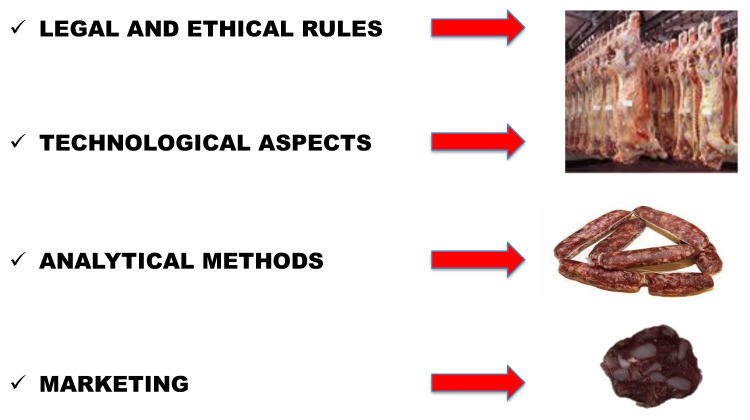
Main issues regarding the quality and authenticity of halal salami.

Traditional ḥalāl meat products can be processed in five different ways: salted and/or marinated but not dried, dried not fermented, fermented semidry/dried, smoked, cooked and/or candied [8]. The production and consumption of ḥalāl meat products, as well as ḥalāl salami, obtained through Islamic slaughter rites, have been growing steadily in Europe over recent decades and even the food safety legislation had to consider religious slaughter practices to avoid conflicts [11]. Salami is one of the most representative dry meat products of Mediterranean countries; it involves long ripening periods, with different final quality due, most of all, to their different compositions as the variable ratio of meat and fat, autochthonous or selected microbiota, additives and ingredients, all of them representative of risks to Ḥalāl assurance [12,13]. Figure 1 depicts the most important issues to be considered when ḥalāl salami products are produced (Figure 1).

The European Union law on slaughter imposes stunning of the animal (applied through electrical, mechanical or gaseous means) before slaughtering, with the exception of ritual slaughter (Regulation EC n. 1099/2009) [14]; for this reason it is important to involve Islamic scholars in the research to enable stakeholders in the meat industry to make decisions regarding the aspects of pre-slaughter stunning [15].

Another aspect that needs to be taken into account is the effect of halal bleeding on meat quality and animal welfare. Aghwan et al. [16] reported that an efficient bleeding process after ḥalāl slaughter not only maintains the quality and wholesomeness of meat but also potentially reduces suffering and pain of animals.

According to the most recent data, it is estimated that about 26 million of Muslims and 1.1 million of Jews live in EU [3], therefore, the number of ritual-slaughtered animals is rather high in Member States [17,18], and this fact requires the particular attention of the Institutions. For example, recently, the General Advocate of the European Court of Justice (ECJ) affirmed that stating that ritual slaughter is incompatible with organic farming this would mean “adding a condition not provided for by current legislation”, while consumers of kosher or ḥalāl products have the right to benefit from the guarantees provided by products labelled “organic farming” in terms of food quality and safety [19].

In Table 1, the rules providing the regulation of ḥalāl foods in Europe are collected. In the present review, the considered rules are those related to safety, hygiene, and quality aspects of ḥalāl salami and other meat products.

## 2. Ḥalāl Salami Processing

Halāl meat is generally consumed as fresh meat, or as processed products, such as pasties, sausages, luncheon meat, turkey breast, bologna or salami.

Generally, salami means fermented and dried sausages manufactured with raw pork, beef or a mixture of pork and beef meat, although other animal species (goat, sheep, goose, horse, donkey, turkey, wild boar) can be used, depending on the typical products and on the geographic area of production [25,26]. Nevertheless, only some of these species could be transformed to halāl salami, and the raw meat must be obtained from ritually slaughtered animals. The different raw materials, as well as the ingredients which should be in compliance with halāl prescriptions raise issues that need to be addressed.

### 2.1. Ḥalāl Raw Material

Ante-mortem treatments and slaughter management could affect carcass traits and meat quality [17,27]. Although most scientists would accept the fact that the meat quality in stunned animals is comparable to that of animals slaughtered without stunning, it has been recently disproved: in fact, when lambs were slaughtered without stunning, their meat developed lower drip before cooking, and had less cooking loss, compared to meat from electrically or CO_2_ stunned lambs [28].

A recent study has demonstrated that three ḥalāl slaughter methods have no substantial effect on lamb meat quality [29], while it has not been tested whether or not ḥalāl slaughter influences the quality of halal dry meat products. The broiled chicken meat sausage was investigated for the effect of ritual slaughter on microbiota: ḥalāl samples were not contaminated with either coliforms, *E. coli* or *Salmonella*, while the non-halal meat sausage contained 1.50 × 10^6^, 2.33 × 10^5^ and 1.50 × 10^5^ CFU g^−1^ of coliforms, *E. coli* and *Salmonella*, respectively [30]. Results of this study highly recommended to follow the Islamic rule in slaughtering poultry and to apply hazard analysis and food hygiene rules to reduce the risk of cross contamination with food-borne pathogens in poultry farms.

Several authors have proposed sheep meat as suitable for ripening processes [31,32,33]; in addition, other studies have been conducted on the influence of animal nutrition, in order to improve the composition quality of meat and fat [34], with a possible influence also on the sensory acceptability and stability of the transformed products.

The use of lard is preferred in batter making (or even dough processing) both for its ready availability and for its functional properties (in particular higher melting point, able to improve organoleptic properties such as texture and succulence), although some limitations exist. In fact, as pork is forbidden in the diet of many people for religious reasons, in ḥalāl salami both meat and fat have to be replaced in agreement with all the other imposed requirements on the manufacture and on the use of ingredients and additives [12]. In Italy, a recent study has collected data regarding market demand for salami with ḥalāl certification; in particular, interviews were carried out with two groups of consumers, of which 103 of Muslim and 151 non-Muslims faith. Both groups of consumers showed a high interest in purchasing equally goat and sheep salami with ḥalāl certification [35]. Unfortunately, some critical issues are related to the replacement of pork meat in dry fermented sausages, due to the low oxidative stability of other fats and the strong sensory impact of the raw materials [26]. Several studies have been conducted on the quality of Turkish sausages made from sheep in order to improve sensory characteristics and texture [36,37], but only few researchers reported on ḥalāl salami: for example, Indian salami, prepared with meat and fat of buffaloes slaughtered according to ḥalāl rites, have been investigated [38]. Furthermore, in ḥalāl salami the influence of spices (es. pepper, paprika, cumin, garlic) and their essential oils, on the inhibition and/or control of alterative phenomena should be considered. For example, during the ripening and storage of dry fermented mutton sausages formulated with pepper and cumin, a significant increase in level of MUFA and PUFA/SFA ratio was observed in respect to the control [39]. Therefore, autochthonous microbiota of fermented sausages could be related to free fatty acids profile as well as to the production of secondary metabolites with toxic action (biogenic amines) [40,41].

To the best of our knowledge, in the literature only few studies declare the preparation of samples according to the ḥalāl procedures, but it would be correct to assume that published researches on Turkish traditional dry-fermented sausage (sucuk) [42,43] concern ḥalāl salami.

Therefore, bez sucuk is a type of Turkish fermented beef sausage, mainly produced by butchers and small-scale facilities that use traditional technologies without adding starter cultures, in which a few manufacturers use curing agents such as sodium nitrite. Bez sucuk differs from other Turkish-type fermented sausages due to the use of cloth casings sewn to size of 7 × 25 cm from uncolored cloth with 42 threads per cm^2^; therefore, the formulation and process conditions (temperature, humidity, and ripening period) show differences among all manufacturers of bez sukuk [44]. Bez sucuk processing has three production steps: mixing the sucuk batter, filling the cloth casings, and ripening for 10–14 days [45].

### 2.2. Preservatives

In general, the effect of the use of additives (glucose, sodium nitrite, sodium nitrate, sodium ascorbate and sodium citrate) on the safety and quality of dry cured meat products has been studied [37,46]. Many chemical ingredients are added in the ḥalāl food production process to enhance the food characteristics, and also preservatives could be added in salami formulation. A halāl food additives checker system has been optimized to provide consumers with a useful result on the product safety meeting Halalan Toyyiban criteria, where the latter indicate that processed foods or ingredients shall be safe for consumption, non-hazardous and non-intoxicating, thus emphasizing quality aspects [47].

In recent years, microorganisms have been a remarkable option for ḥalāl production. Halāl principles must be followed in the manufacturing of bioproducts, therefore the adding of microbial ingredients must match this specification too. Assuming that in the future the share of ḥalāl microbial products will increase in the biotechnology market, Karahalil et al. [48] evaluated the steps of a fermentation process from an Islamic point of view and determined the control points for ḥalāl requirements.

### 2.3. Sensory Profile

The sensory characteristics of processed meats could be affected by several factors, such as the kind and the quality of the raw material, the ingredients (other than meat), the eventual addition of starter cultures and the processing. As the raw materials are different from classical sausages, their impact on the final sensory traits of the product has to be considered.

Recently, different percentages of mutton (from adult female sheep, over four years old) and additional autochthonous starter cultures (*Staphylococcus xylosus* LQ3 and *Pedioccoccus pentosaceus* P38) were tested in a study on fermented Turkish sausages [49]. The results of this research showed that the use of indigenous microbial cultures attributed positive and typical characteristics to the fermented sausages, with a high hedonic score for sensory acceptance; furthermore, a positive effect of mutton on the reduction of unsaturated fatty acids and an increase in red tonality were proved.

Moreover, the volatile profile of fermented meat sausages containing 90% of mutton was characterized by a higher abundance of butyric (C4: 0), hexanoic (C6: 0) and octanoic (C8: 0) acid, related to hydrolytic rancidity or to the oxidation of fatty acids; moreover, butanal and a high level of hexanal were detected, too. Generally, low concentrations of short chain saturated fatty acids (up to 10 carbon atoms) are desirable in fermented meat [50]; furthermore, autoxidation of long chain unsaturated fatty acids can generate aldehydes and other aliphatic volatile compounds [51].

With the aim of meeting the growing need for meat in developing countries, several research projects have been carried out on new formulations of ḥalāl salami, such as sucuk reformulated with camel meat and hump fat [52]. The camel meat, especially from young animals, contains less fat and cholesterol and relatively higher PUFA than other meats; therefore, camel-hump fat is used for the production of a cocoa-butter analogue, so its use in dry sausages provides final products of high-quality. In fact, results of this investigation showed a good potentiality of these innovative raw materials, such as sucuk made from camel meat and hump fat showed physical-chemical, fatty-acid and volatile-compounds profiles and sensory qualities similar to sausages made from beef and beef fat (traditional sukuc).

### 2.4. Biogenic Amines

Biogenic amines have been implicated as the causative agent in several food poisoning outbreaks. Fermented food, such as Turkish style fermented sausages, can also contain biogenic amines; in fact, microorganisms possessing the enzymes and amino acids decarboxylases, which convert amino acids into biogenic amines, are responsible for the formation of these compounds in fermented meats. In addition, in dry fermented mutton sausages, safety and quality have been proved to be difficult to guarantee, particularly because of the presence of biogenic amines, which can accumulate, as a consequence of the presence of producing bacteria [53]. High concentrations of BAs have been found in industrial dry sausages added with starter cultures and not only in artisanal ones, because pure or starter cultures could not be sufficiently competitive in suppressing the growth of wild amine-producing microbiota [41]. Thus, the quality of the raw materials and ingredients and the hygienic processing practices are crucial to control the BAs production in fermented meat products; nevertheless, selected starter cultures could also help in containing the BAs amount. In fact, although the amino acids decarboxylase potential is strain specific, starter species such as *Lactobacillus sakei, Lb. plantarum* and *Staphylococcus xylosus*, are generally described as weak or non-aminogenic bacteria. Moreover, different studies have been conducted to evaluate the effect of a combination of negative amine producer starter cultures (*Lactobacillus* spp., *Pediococcus* spp., *Staphylococcus* spp. and *Micrococcus* spp.) in the reduction of the biogenic amines amount during fermented sausages manufacture, with interesting results, proving a BAs reduction from 9% up to about 100%, depending on the specific biogenic amine [54]. These studies underline the importance to test the starter culture strains with the aim of improving the quality and safety of the final product.

As a whole, the sum of vasoactive biogenic amines (tyramine, histamine, tryptamine, 2-phenylethylamine) results not exceeding 200 mg kg^−1^ when dry fermented sausages have been manufactured according to excellent hygienic conditions and good manufacturing practices (GMP) [55].

Ekici and Omer [56] investigated the biogenic amines concentration reached in 120 sukuc samples collected from 10 different brands sold in the local markets of Van (Turkey). Tryptamine (0–129.4 mg/kg), 2-phenylethylamine (0–65.6 mg/kg), putrescine (0–255.6 mg/kg), cadaverine (0–1148.8 mg/kg), histamine (0–469.4 mg/kg), tyramine (0–438.1 mg/kg), spermidine (0–554.4 mg/kg) and spermine (0–614.4 mg/kg) were detected, showing that the occurrence of biogenic amines represent a real risk associated with the fermentation of ḥalāl salami.

Other studies were carried out on bez sucuks produced with different meat:fat ratios (90:10, 80:20 and 70:30, respectively); the results showed that bez sucuks with the highest meat ratio (90:10) had the highest tryptamine, putrescine, and tyramine levels at the end of the processing and storage period [57].

Spices and other plant materials used in fermented meat for their flavoring effect, as well as for the antioxidant and bacteriostatic activity, due to the content in essential oils, phenolic compounds and organic acids, can also reduce the formation of biogenic amines [42,58] (see next section).

### 2.5. Use of Spices and/or Plant Extracts

Often added to fermented meat products with the aim of enriching the taste and the sensory characteristics, spices and plant extracts also exert interesting bioactivities. In detail, the phenolic constituents of spices and plant extracts are able to interact with the cytoplasmic membrane modifying its fluidity and permeability [59] up to the rupture, with consequent impairment of energy production and leakage of cytoplasmic material [60]. These effects could be useful in contrasting the viability and the metabolic activity of biogenic amines-producing bacteria, with greater effects than nitrites [53]. For example, Jia et al. [53] investigated the inhibitory effect of several spices including clove, cassia, bay leaf, fennel, star anise and nutmeg on the biogenic amines accumulation in dry fermented mutton sausages, revealing that particularly cassia and fennel were very effective in reducing the biogenic amines amount. In detail, reductions up to 27.5% were observed for spermidine, followed by 24.6% for 2-phenylethylamine, 21.8% for tryptamine, 18.7% for tyramine and even 24.4% for histamine, thus proving the importance of spices for the safety of fermented meat products, at least from this point of view.

Nevertheless, spices were demonstrated to improve also the safety profile of pastrami, a dry-cured meat product traditionally produced in Egypt with beef, lamb, water buffalo or camel meat, and very common also in Mediterranean and Middle East countries [61]. In detail, spices contained in a seasoning paste made of salt, sweet and hot pepper, fresh garlic, clove, coriander, rosemary, fenugreek seeds and nutmeg, decreased *Escherichia coli* and aerobic microbial counts and reduced aflatoxins content below the permission limit of 20 ppb [62].

Due to their antioxidant activity, spices such as curry leaves, torch ginger and cinnamon have been proved to maintain the quality of lamb meat also during cooking processes, reducing the formation of heterocyclic aromatic amines, poly aromatic hydrocarbons and trans fatty acids [63]. In 2015, the International Agency for Research on Cancer from the World Health Organization, recognized the above mentioned compounds as responsible for cancerogenicity for consumption of red meat and processed meat. An overview of the effectiveness of spices and natural products in counteracting the development of potential carcinogenic substances in meat products has been recently provided by Lee et al. [64].

Most of all, spices in meat products are essential to contain the oxidative reactions at the expense of lipid and protein fractions, leading to pigment, flavor, and texture deterioration and to the shelf-life reduction. As raw materials often rich in unsaturated fatty acids are used to produce ḥalāl fermented meat products, the role of spices in this kind of product is particularly important to improve the oxidative state of the final product. Mediterranean plants exert antioxidant activity due to the presence of phenolic compounds, terpenes, organosulfur compounds, acids and other molecules, able to contrast proteins and lipids oxidation, decreasing metal ions and scavenging radicals [65]. The same chemical species allow the spices to exert antibacterial and fungicidal activity, against spoilage and pathogenic microorganisms, acting as biopreservatives, improving the safety profile and extending the shelf-life of processed meat products. Therefore, although they are traditionally used in meat and meat products to enrich and enhance the sensory profile of the products, spices and plant extracts have an important impact on many aspects of the products, thus protecting the consumers health, and resulting in a clean label also for ḥalāl meat products [66]. Necessarily, for ḥalāl products, the spices must be ḥalāl-suitable and particular attention has to be paid to spices blends, where non-certified animal-based ingredients have to be avoided, and the risk of cross-contamination should be carefully checked.

### 2.6. Halāl Casing

Currently, the use of ḥalāl meats increases the request for ḥalāl casings. In fact, while the traditional pork casings are obviously forbidden, those obtained by other animals are allowed, as long as these animals have been slaughtered in compliance with ḥalāl provisions. Moreover, beside non animal casings such as those made of cellulose and other plant materials, innovative solutions are actually under study. For example, the production of a chitosan casing could be well-suited for commercial application in ḥalāl sausages. A study on a novel chitosan-based casing provided an alternative packaging material to collagen to be used as a sausage casing for the meat industry, showing similar mechanical properties as the collagen casing, but lower water solubility, superior transparency, and better UV light barrier [67]. Recently Marcos et al. [68] proposed the co-extruded alginate coating as a feasible alternative to collagen casing: in fact they observed a regular evolution of pH values during the fermentation step (from the initial value about 6.0, the pH decreased just below 5.0) and the control of spoilage microorganisms; no significant difference resulted on the final a_w_ value (<0.92), but a faster drying kinetic was observed in sausages with alginate coating compared with the ones stuffed into collagen casings; finally, authors reported no significant differences on the sensory properties between different casing types.

Sezer and Bozkurt [43] tested the applications of active packaging on the stability of traditional Turkish type fermented sausage; these authors carried out a study concerning the effect of the incorporation of antimicrobials (chitosan and silver substituted zeolite, AgZeo) into multilayer films as a novel casing. Chitosan has an antimicrobial spectrum against Gram(+)/Gram(–) bacteria, molds, and yeasts [69], whereas Ag-ions exert high antimicrobial activities due to their inactivation effect towards a series of metabolic enzymes [70].

Aerobic plate count and lactic acid bacteria were decreased significantly (*p* < 0.05) by chitosan-incorporated casing; moreover, antimicrobial plastic casings including chitosan and AgZeo decreased (*p* < 0.05) putrescine, histamine, and tyramine formation in sucuks, therefore, these novel casings could be used to improve quality and safety of ḥalāl salami [43].

Finally, a very important aspect to be studied is the evolution of dehydration processes to assess the diffusive phenomena of salt and water, in order to build simple predictive models concerning the safety and quality of ḥalāl salami and other ḥalāl cured meat products [71].

## 3. Food Safety in Ḥalāl Assurance

Nowadays food safety is a responsibility of government agencies and organizations. European Community (EC) legislation (see Table 1) is primarily geared towards ensuring the production of safe foods for human health but also for ensuring free competition in the food market.

For foodstuffs of animal origin, further specific hygiene requirements are necessary (prescribed by Reg. 853/2004) [22], as these products may present macrobiotic and chemical risks for the human health and therefore, they require the application of specific rules. The rules dictated by Reg. 853/2004 are added to those related to animal welfare [72], without posing particular issues to ritual slaughter operators.

In addition to the above mentioned laws, Regulation (EC) N. 1935/2004 of the European Parliament and of the Council of 27 October 2004 on the regulation of materials and articles intended to come into contact with foodstuffs [23] should also be taken in account. In general, once these materials come into contact with food, they must not cause unacceptable changes in foodstuffs; nor should these provisions pose particular problems to ḥalāl slaughter operators.

Hazard Analysis and Critical Control point (HACCP) system is considered to be effective for enhancing food safety; furthermore, other standards, such as ISO series, Approved Quality Assurance (AQA), Good Agricultural Practices (GAP), Good Manufacturing Practices (GMP) and the Food Safety Management System (FSMS) could be considered for food quality and safety. These standards could be contemplated by more than one hundred active certifying bodies, governmental or non-governmental organizations, for ḥalāl compliance [73]. In a recent study, many areas for potential research in halāl assurance in the food industry have been identified, and critical issues have been highlighted [74]. Incorporating halāl features into the HACCP system could be a plausible tool for halāl assurance. As the HACCP and Ḥalāl certification processes are similar, the integration of the halāl assurance scheme into the HACCP system could be feasible [75].

Although the knowledge of all factors influencing the ḥalāl assurance systems is a determinant to help companies to identify intervention strategies to improve their performance, limited literature is available on this issue [76]. Recently, Malaysian researchers have applied an interesting study design to explore critical factors affecting the ḥalāl assurance systems: different factors in every country, region or food chain can be found, with differences resulting between food sectors and subsectors and among small or medium (SME) and large-sized enterprises [77]. These studies are particularly important for SMEs that have limited resources.

## 4. Authentication of ḥalāl Meat for Salami and Other Meat Products

With the increasing population, the demand for ḥalāl food products also increases, putting a responsibility upon government, jurisprudence and companies to certify ḥalāl products [78].

The matching of each product with the label statement is a quality requirement; in European countries it is mandatory that the products are labelled in accordance with Regulation (EC) n. 1169/2011 of the European Parliament and of the Council of 25 October 2011 on the provision of food information to consumers [24]. Furthermore, for ḥalāl foods, the need for clear ḥalāl labels, ensuring that the product (from the ingredients to the processing and handling) meets the appropriate requirements, is a critical issue. Due to the repeated discoveries of non-ḥalāl ingredients in food otherwise labelled as ḥalāl, the status concerning the determination of ḥalāl and non-ḥalāl food products needs to be carefully read.

The analytical authentication of ḥalāl foods has the purpose of solving problems such as fraud and adulteration of ḥalāl products that are highly critical both for importing countries (such as Malaysia, Saudi Arabia, Singapore and Brunei Darussalam) and for the top ḥalāl food exporters (Brazil, Australia, USA and France) [79,80,81]. Various ḥalāl supervision agencies work closely with food industries to obtain the permission to use their supervision agency’s trademark symbol on their products.

Ḥalāl authenticity is an issue of major concern in the food industry, and methods of lard detection have been performed for the investigation in food products such as cakes and chocolate [82,83]. Moreover, specific techniques able to exclude their possible contamination or fraud have been developed [84,85,86]. A frequent adulteration of meat products is the addition of pork to beef products, which is carried out for economic gain and represents a serious problem in the halāl food industry, in particular for the authenticity of minced and homogenized meat products. Moreover, often companies producing halāl meat products also process other kinds of meat, and thus cross-contaminations are possible. Therefore, many researches have been recently carried out for authenticating the species composition of meat products [87,88,89,90,91] and in particular the ḥalāl authentication studies are focused on the detection of pork derivatives (meat or lard) [92,93].

Different commercial kits which investigate porcine protein and DNA have been developed in many countries (such as USA, UK, France and Belgium) in order to establish the ḥalāl authenticity of food products [94]; kits are useful as they generally allow a rapid determination of the contamination, nevertheless the addition of meat different from pork could remain undetected. To date, different techniques are routinely applied for meat species detection and identification in food: in Table 2 and Table 3, protein-based and genetic methods suitable for evaluating the authenticity of halāl meat products are reported, respectively. Due to the characteristic of proteins that tend to denature at high temperatures, these methods have limitations in the detection of animal species from cooked, baked or heat-treated food products; on the contrary, DNA-based methods are more sensitive and reliable, as DNA is found in a majority of cells, it is species-specific and is stable at higher temperatures [95]. However, meat processing could denature short DNA sequences [96] whilst the primary structure of peptides is relatively stable; for this reason a possible approach for highly processed meat authentication could be the combination of chromatography with mass spectrometry (MS), thus investigating the molecular weight and amino acid sequence of meat proteins [13].

The PCR amplification of pork mitochondrial genes (12S and 18Sribosomal RNA subunits and cytochrome b) and of the displacement loop region (D-loop) was successfully applied for the detection of pork derivatives and was found to be a suitable technique for routine food analysis and halāl certification [115].

In 2018, for the first time a tetraplex polymerase chain reaction-restriction fragment length polymorphism (PCR-RFLP) assay to identify and discriminate rabbit, rat and squirrel meat in frankfurter formulation was developed and validated. The detection limit of the assay was 0.1% meat in frankfurter formulation. Moreover, results shown in this research assessed that variations in food processing treatments could not affect the stability of the optimized assay [92]. However, it should be considered that it is rather unlikely that rabbit or squirrel meat can be used as a substitute for chicken or beef, because their meat is certainly more expensive.

Nevertheless, although classical PCR and real time-PCR are the most frequently used methods, they usually target a limited pool of species, and usually the most used, such as pork, beef, horse and chicken, whereas the potential adulteration with exotic species often remains uncovered. For this purpose, Cottenet and colleagues [117] have recently developed a Next Generation Sequencing method for the identification of different species, both in pure meat samples, with optimal results, and in mixtures, where the species were correctly identified in spiked samples down to 1% (*w*/*w*). Together with the most common species, also yak, donkey, zebra, hare, fallow deer, reindeer, muskrat, fox, weasel, dog, cat pigeon and rat, and up to 46 different species were detected, amplifying and sequencing a mitochondrial DNA fragment of about 120 bp. Unfortunately, the method was less effective when applied to ground meat, suggesting that work is still required to improve the results.

Moreover, Lavelli has suggested a scheme of traceability implementation for the poultry meat supply chain: the author presented a case study to discuss both the advantages and difficulties of setting up a high-warrant traceability procedure conform to “generic” or “specific” traceability systems, depending on many different factors (technological and economical aspects, specific regulations and internal objectives) [125].

The genetic technologies, such as simple sequence repeat (SSR) and single nucleotide polymorphism (SNP) can be very reliable in the traceability management system of ḥalāl foods [126,127]. Some studies were carried out in order to select SNPs panel useful for traceability of ḥalāl beef [81,110].

A recent study reported about a particular pork peptide (signature sequence LVVITAGAR, from lactate dehydrogenase) that was not recorded in other meats; in this study, liquid based chromatography coupled with mass spectrometry (LC-MS) was used for the detection of the identified porcine-specific peptide as a thermostable marker of highly processed haram (that means proscribed by the Islamic law) meat products [99].

Interestingly, proton nuclear magnetic resonance (^1^H-NMR) has been applied together with HPLC as a high-performance approach to detect lard used to adulterate butter. In fact, the triacylglycerol (TAG) composition of lard can be used as chemical marker for ḥalāl authentication. More precisely, peaks recorded in the region of 2.60–2.84 ppm highlight specific characteristics present only in lard, and these frequencies can be considered specifically for the analysis [128].

**Figure 2:**
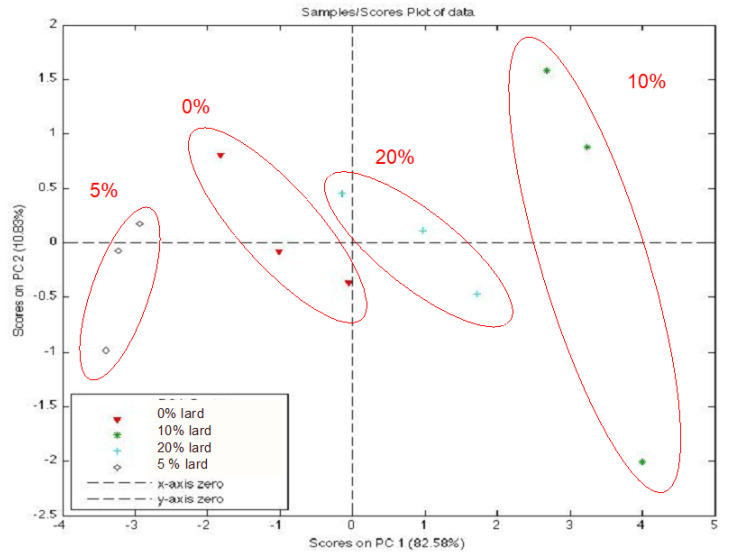
Principal component analyses (PCA) scores on porphyrins-based EN analysis on ḥalāl goat meat dough added with pork fat (0%, 5%, 10% and 20% of lard, *w*/*w*).

A rapid, accurate, convenient, and eco-friendly analysis for the detection of porcine-based ingredients in food is the Electronic Nose (EN), employed to exclude possible contamination or fraud and also to investigate the oxidative status of meat products [85,86,101,102]. The ability to measure and identify the aroma and characteristics of persistent flavors of a product allows to obtain a variety of information, directly related to the acceptability and to nutritional and quality characteristics in terms of product health and safety. This consideration has led to a growing need and interest in non-destructive monitoring systems with high versatility, sensitivity, accuracy, cost-effectiveness, ease of use and above all rapidity of analytical response, such as electronic nose. This artificial olfactory systems can be used not only in the laboratory but also directly in the production plants for continuous monitoring of odors, from the raw material to the final product, analyzing the volatile compounds released from the matrix related to different aspects (sensory acceptability, deterioration, development of off-flavors, etc.). In our laboratories an electronic nose equipped with non-specific sensors arrays (porphyirins based) has been used to discriminate the lard presence in ḥalāl goat meat batter. First, four experimental batches of salami were manufactured with halal goat meat (70%) and fat (30%); each of the four batches had different levels of pork fat (batch 1, Control, with lard 0% *w*/*w* of total fat; other batches, with lard 5%, 10% and 20% *w*/*w* of total fat, respectively); then EN analyses were carried out after an equilibration step (for 30 min, at temperature of 41 °C). Principal Component analyses (PCA) scores (Figure 2) evidenced that porphyirins-based EN was able to discriminate the presence of lard even at the lowest experimental concentration (5%, *w*/*w*) [129].

Furthermore, a gas sensor array based on peptide modified gold nanoparticles deposited onto 20-MHz quartz crystal microbalances [102] has been also applied to discriminate the lard presence in ḥalāl meat products and investigate the shelf life of ḥalāl dry meat sausages (trials are still going on; unpublished data).

Finally, new approaches regarding ḥalāl authentication, including the latest biotechnological innovations, such as assays and the use of smartphones, are being also developed [130,131].

## 5. Conclusions

The quality assessment and authentication of ḥalāl products are issues raising a growing interest in European Community Countries (France, Sweden, Germany, Greece, Spain, Italy), Switzerland, Russia and other countries in the world (Asia, the UK, South and North America).

Considering that top producers of ḥalāl products (including meat) are countries where Muslims are a minority, future research should take into consideration ḥalāl standards, immigration and integration of qualified Muslim workers, as evidenced by a study recently carried out in Brazil [132]. As regards specifically halāl salami, the origin of the animal raw meat, as well as ingredients and additives are the main concerns for consumers of Islamic faith. The pork matrix may be fraudulently present in processed meat, for both economic and technological purposes. In fact, the use of these raw materials (which are easier to find) has a lower cost; furthermore, it allows manufacturers to obtain final cured meat with better quality (sensory properties) and stability (especially with respect to oxidative reactions).

Many approaches have been proposed in the literature for the evaluation of the authenticity of salami and other meat products with ḥalāl certification. The research of accurate analytical methods for the differentiation of meat species is therefore of great importance for both companies and consumers, and important advances have been made in recent years, while analytical methods to distinguish the type of slaughter applied to obtain the meat are still difficult to optimize. Moreover, the literature is often focused on ḥalāl meat products, while comparisons with non ḥalāl analogue products are still scarce or even missing. Future researchers should carry out further studies on ḥalāl food in order to provide useful information about major factors related to quality and stability, especially for ḥalāl dry cured meat products, such as salami. The market for ḥalāl products is evolving, considering that the non-Islamic consumer would seem to associate the ḥalāl brand with “superior” quality [133]. This would be very important to properly orient the companies that would like to diversify their production and also to guarantee food safety and consumer satisfaction.

## Figures and Tables

**Table 1:** Rules providing the regulation of ḥalāl foods in Europe.

Subject	Law	Regulation Issue
Food safety	Regulation (EC) n. 178/2002 [20]	on laying down the general principles and requirements of food law, establishing the European Food Safety Authority and laying down procedures in matters of food safety;
Food hygiene	Regulation (EC) n. 852/2004 [21]	on the hygiene of foodstuffs;
	Regulation (EC) n. 853/2004 [22]	laying down specific hygiene rules for food of animal origin;
Food contact materials	Regulation (EC) n. 1935/2004 [23]	on materials and articles intended to come into contact with food;
Animal slaughter (including ritual one)	Regulation (EC) n. 1099/2009 [14]	on the protection of animals at the time of killing;
Label statement	Regulation (EC) n. 1169/2011 [24]	on the provision of food information to consumers;

**Table 2:** Examples of protein-based methods for ḥalāl authenticity analysis.

Methods	Aim	References
*Immunoassay (ELISA)*	Porcine gelatin determination in processed foods	[97]
*Isoelectric focusing (IEF)*	Meat authentication in raw and cooked meat products	[98]
*Chromatography and mass spectrometry (MS)*	Meat authentication	[13,79]
	Meat species determination	[99]
*Electric nose (EN)*	Pork fat detection	[100]
	Pork meat detection	[66]
*Mass spectrometry soft ionization*	Identification of muscle proteins of different species	[101]
	Horse and pork meat detection	[102]
	Horse and pork meat detection in highly processed food	[103]
*Fourier transform infrared spectroscopy (FTIR);*	Lard detection	[104]
	Pork detection in sausages	[105,106]
*Near-infrared spectroscopy (NIR; FT-NIR)*	Pork derivatives detection	[107,108]
	Adulteration of meat	[109]

**Table 3:** Examples of genetic methods for ḥalāl authenticity analysis.

Methods	Aim	References
*Simple sequence repeat (SSR) and Single nucleotide polymorphism (SNP)*	Meat traceability Meat fraud	[81] [110]
*Polymerase chain reaction (PCR)*	Pork derivatives detection Pork derivatives detection in gelatin Meat species identification Authenticity determination	[11,84] [111] [85,112,113] [94]
*PCR- Restriction Fragment length polymorphism (PCR-RFLP)*	Pork meat detection in meat products Rabbit, rat and squirrel meat detection in frankfurter	[114] [92]
*Real Time PCR*	Horse and donkey meat detection Species identification of meat Pork meat detection Pork meat detection and quantification	[115,116]
*Next Generation Sequencing*	Identification of 46 different meat species in pure samples, in spiked samples and in ground meat samples	[117]
*Aptamers*	Application in analysis of foods	[118]
*Isothermal amplification*	Meat species identification Detection of meat of different species Review on Isothermal amplification techniques Rapid on-site detection of meat pork Nucleic acid detection	[119] [120] [121] [122] [123,124]
